# PFAS and Phthalate/DINCH Exposure in Association with Age at Menarche in Teenagers of the HBM4EU Aligned Studies

**DOI:** 10.3390/toxics11080711

**Published:** 2023-08-18

**Authors:** Bianca Cox, Natasha Wauters, Andrea Rodríguez-Carrillo, Lützen Portengen, Antje Gerofke, Marike Kolossa-Gehring, Sanna Lignell, Anna Karin Lindroos, Lucia Fabelova, Lubica Palkovicova Murinova, Anteneh Desalegn, Nina Iszatt, Tessa Schillemans, Agneta Åkesson, Ann Colles, Elly Den Hond, Gudrun Koppen, Nicolas Van Larebeke, Greet Schoeters, Eva Govarts, Sylvie Remy

**Affiliations:** 1 VITO Health, Flemish Institute for Technological Research (VITO), Boeretang 200, 2400 Mol, Belgium; natasha.wauters@vito.be (N.W.); andrea.rodriguezcarrillo@vito.be (A.R.-C.); ann.colles@vito.be (A.C.); gudrun.koppen@vito.be (G.K.); greet.schoeters@uantwerpen.be (G.S.); eva.govarts@vito.be (E.G.); sylvie.remy@vito.be (S.R.); 2 Toxicological Centre, University of Antwerp, Universiteitsplein, 1, 2610 Wilrijk, Belgium; 3 Institute for Risk Assessment Sciences, Utrecht University, 3584 Utrecht, The Netherlands; l.portengen@uu.nl; 4 German Environment Agency, Umweltbundesamt (UBA), 14195 Berlin, Germany; antje.gerofke@uba.de (A.G.); marike.kolossa@uba.de (M.K.-G.); 5 Swedish Food Agency, 751 26 Uppsala, Sweden; sanna.lignell@slv.se (S.L.); annakarin.lindroos@slv.se (A.K.L.); 6 Department of Environmental Medicine, Faculty of Public Health, Slovak Medical University, 831 01 Bratislava, Slovakia; lucia.fabelova@szu.sk (L.F.); lubica.murinova@szu.sk (L.P.M.); 7 Division of Climate and Environmental Health, Norwegian Institute of Public Health, 0456 Oslo, Norway; a.a.desalegn@farmasi.uio.no (A.D.); ninalouise.torcelino-iszatt@fhi.no (N.I.); 8 Unit of Cardiovascular and Nutritional Epidemiology, Institute of Environmental Medicine, Karolinska Institutet, 171 77 Stockholm, Sweden; tessa.schillemans@ki.se (T.S.); agneta.akesson@ki.se (A.Å.); 9 Provincial Institute of Hygiene, Provincial Research Centre for Environment and Health, 2023 Antwerp, Belgium; elly.denhond@provincieantwerpen.be; 10 Analytical, Environmental and Geo-Chemistry, Vrije Universiteit Brussel, 1050 Brussels, Belgium; nicolas.van.larebeke@vub.be; 11 Department of Biomedical Sciences, University of Antwerp, 2000 Antwerp, Belgium

**Keywords:** PFAS, phthalates, DINCH, age at menarche, HBM4EU, multi-pollutant models

## Abstract

Early puberty has been found to be associated with adverse health outcomes such as metabolic and cardiovascular diseases and hormone-dependent cancers. The decrease in age at menarche observed during the past decades has been linked to an increased exposure to endocrine-disrupting compounds (EDCs). Evidence for the association between PFAS and phthalate exposure and menarche onset, however, is inconsistent. We studied the association between PFAS and phthalate/DINCH exposure and age at menarche using data of 514 teenagers (12 to 18 years) from four aligned studies of the Human Biomonitoring for Europe initiative (HBM4EU): Riksmaten Adolescents 2016–2017 (Sweden), PCB cohort (follow-up; Slovakia), GerES V-sub (Germany), and FLEHS IV (Belgium). PFAS concentrations were measured in blood, and phthalate/DINCH concentrations in urine. We assessed the role of each individual pollutant within the context of the others, by using different multi-pollutant approaches, adjusting for age, age- and sex-standardized body mass index z-score and household educational level. Exposure to di(2-ethylhexyl) phthalate (DEHP), especially mono(2-ethyl-5-hydroxyhexyl) phthalate (5OH-MEHP), was associated with an earlier age at menarche, with estimates per interquartile fold change in 5OH-MEHP ranging from −0.34 to −0.12 years in the different models. Findings from this study indicated associations between age at menarche and some specific EDCs at concentrations detected in the general European population, but due to the study design (menarche onset preceded the chemical measurements), caution is needed in the interpretation of causality.

## 1. Introduction

Puberty is characterized by several processes that lead to sexual maturation and the attainment of reproductive capacity. Since the end of the 19th century a considerable decrease in the age of pubertal onset has been observed, especially among girls [1]. The trend for earlier onset of menarche (first menstrual bleeding) and earlier development of secondary sexual characteristics has coincided with improved public health and nutrition [2]. While the age at menarche was thought to have stabilized in the past 50 years, recent studies suggest that the decrease is ongoing [3,4,5,6]. This trend toward earlier puberty is of considerable concern, because altered pubertal timing has been linked with adverse health outcomes in adolescence and adulthood [7]. Menarche normally occurs between the ages of 10 and 16 and generally around 12–13 years [1,2]. Earlier menarche has been associated with type 2 diabetes [8], cardiovascular disease [9], asthma, and reproductive cancers [10,11]. Early as well as late menarche has been associated with psychological and behavioral disorders [7].

The decrease in age at menarche has partially been attributed to the increasing prevalence of childhood obesity because, in addition to genetic factors, energy availability and adiposity play an important role in pubertal timing [1,12]. The earlier onset of puberty has also been linked to environmental exposure to endocrine-disrupting compounds (EDCs) [2,13,14,15,16]. EDCs may have direct effects on puberty and reproduction, but also indirect effects through the control of energy balance, meaning that the timing of puberty could be affected by an interplay between obesity and EDC exposure [17].

Per- and polyfluoroalkyl substances (PFAS) and phthalates are two important classes of EDCs that can be found in a wide spectrum of industrial and consumer products, such as food packaging, toys, clothing, and personal care products. PFAS are commonly used to make products resistant to water, oil and grease, whereas phthalates are used as softeners to make plastics more flexible. Their widespread use has caused severe contamination of soil, water and food, resulting in harmful exposure to humans, which can happen through digestion, dermal absorption, and inhalation [18,19]. Several phthalates, including di(2-ethylhexyl) phthalate (DEHP), are reproductive toxicants of category 1B of Annex VI of the Classification, Labeling and Packaging (CLP) regulation [20] and are on the REACH Candidate List of Substances of Very High Concern (SVHC) for Authorisation [21]. DEHP, dibutyl phthalate (DBP) and benzyl butyl phthalate (BBP) were restricted for use in toys and childcare products in concentrations >0.1% weight plasticized material in 2005 [22], and di-isononyl phthalate [DINP], Di-isodecyl phthalate (DIDP) and di-n-octyl phthalate (DNOP) [23] in 2009. The phthalate substitute di-iso-nonyl-cyclohexane-1,2-dicarboxylate (DINCH; Hexamoll^®^) was introduced in 2002 to replace many of the higher molecular weight phthalate esters in food packaging materials, medical devices, and children’s toys [24], because it was assumed to have a less hazardous toxicological profile compared to phthalates [25,26].

PFAS and phthalates have been found to alter steroidogenesis, impact the hypothalamic–pituitary–gonadal (HPG) axis and interfere with the intracellular signaling components of the endocrine system [17,27]. PFAS and phthalate exposure has been linked to several adverse health effects, including metabolic diseases, asthma, immunotoxicity, adverse birth outcomes, and reproductive and fertility issues [17,28,29,30,31,32]. They can also pass through the placenta and interfere with embryonic and fetal development [17,33]. Epidemiological evidence for adverse health effects of DINCH is limited, but in vitro and in vivo studies have shown that the DINCH metabolite monoisononylcyclohexane-1,2-dicarboxylic acid ester (MINCH) is a PPAR-α, PPAR-γ, Er-α, Er-β and AR agonist [34,35,36] and a metabolic disruptor [35]. DINCH can cause oxidative stress in human THP-1 macrophages [37] and impact steroidogenesis [38,39,40], hepatic gene expression, Leydig cell function, DNA replication pathways and liver metabolic capacity [41].

Evidence for effects of PFAS and phthalate exposure on timing of puberty is inconclusive [13,33,42,43], with some epidemiological studies reporting early puberty [44,45,46] and others delayed puberty [45,46,47,48,49,50,51] in association with increased exposure to these EDCs. Differences in sex, compound properties, concentration, co-exposures, and window of exposure assessment may explain inconsistent findings across studies. The majority of studies examined exposure to one EDC at a time, thereby ignoring potential confounding by other substances in the mixture and not accounting for potential mixture compositions in which the presence of certain compounds may alter the activity of others. In addition, more recently introduced phthalate alternatives such as DINCH are understudied.

We investigated the association between biomarkers of exposure for PFAS and phthalates/DINCH and age at menarche in four European studies. The harmonized analyses of this study were embedded in the European Human Biomonitoring initiative (HBM4EU), a project co-financed under Horizon 2020, aiming at the coordination, harmonization, and advancement of human biomonitoring to enable science-based chemical policy development [52]. We used four widely applied statistical methods for studying health effects of chemical mixtures for which user-friendly software packages are available, including different classes of approaches (frequentist, Bayesian, model selection, penalization, etc.) with a different degree of flexibility and computational efficiency: ordinary linear single- and multi-pollutant regression models, Bayesian model averaging using Bayesian adaptive sampling, elastic net, and Bayesian kernel machine regression.

## 2. Materials and Methods

### 2.1. Study Population

The study population was part of the HBM4EU aligned studies, which were set up to collect harmonized and quality-controlled data on recent internal exposure to environmental pollutants in the European population [53,54,55]. The HBM4EU aligned studies focused on children (6–11 years), teenagers (12–19 years) and adults (20–39 years), aiming at geographical coverage by recruiting participants from the four geographical regions of Europe (north, east, south, and west). The number of participants within each country was limited to a maximum of 300 per age group, and an approximate ratio of 50/50 male and female participants was required. Detailed information about the process of study selection and data homogenization has been described previously [53,54]. The participating aligned studies had to fulfill the following criteria: (1) have collected questionnaire data, (2) have available biological samples collected within the period 2014–2021, and (3) have signed informed consent from the participant and/or from legal guardian(s) when the participant was younger than 16 years.

PFAS, phthalates and DINCH (Hexamoll^®^) were measured in nine out of the eleven aligned studies in teenagers, of which five also had data on age at menarche available: Riksmaten adolescents 2016–2017 (Sweden; age range 12–17 years), PCB cohort follow-up (Endocrine disruptors and health in children and teenagers in Slovakia; Slovakia; 15–17 years), Flemish Environment and Health Study IV (FLEHS IV; Belgium; 13–16 years), Cross-Mediterranean Environment and Health Network (CROME; Greece; 12–17 years), and German Environmental Survey, 2014–2017 unweighted subsample (GerES V-sub; Germany; 12–18 years). All studies were cross-sectional, except for the PCB birth cohort in which mothers were recruited at delivery and their children were followed up over time. Only cross-sectional data from the follow-up investigation at 15–17 years were used in this study. Riksmaten adolescents 2016–2017, FLEHS IV, and GerES V-sub included 150 female teenagers each, of which 130 (86.7%), 136 (90.7%), and 117 (78.0%) had reached menarche according to the binary menarche status variable, respectively. The PCB cohort follow-up contained 169 female teenagers, of which 159 (94.1%) had data on age at menarche (the menarche status variable was not available for this study). Because of the low number of participants with data on age at menarche in the CROME study (75 female teenagers, of which only 45 had reached menarche), this study was excluded from our analysis.

### 2.2. Chemical Analysis

Chemicals were measured by liquid chromatography-tandem mass spectrometry (LC-MS/MS), except for PFAS data from Riksmaten adolescents 2016–2017, which were obtained by ultraperformance liquid chromatography-tandem mass spectrometry (UPLC-MS/MS). Chemical measurements were rated quality assured by the HBM4EU quality assurance quality control (QA/QC) program [56,57], except for OH-(mono-isononyl) phthalate (OH-MiNP) measurements from the PCB cohort follow-up and for PFAS and mono-ethyl phthalate (MEP) measurements from Riksmaten adolescents 2016–2017. The Swedish laboratory presented PFAS measured in Riksmaten adolescents in ng/g (μg/kg), which was reported to HBM4EU in μg/L assuming that 1 mL blood serum equals 1 g blood serum. All studies measured PFAS concentrations in serum and phthalates and DINCH in spot urine, except for GerES, which provided plasma and first morning urine measurements, respectively. As the concentrations in urine are influenced by dilution level, specific gravity was used to adjust for the urinary dilution by normalizing samples to a standard or a population average urinary concentration of 1.024 [58].

All laboratories reported limits of quantification (LOQs), which varied among the studies), except for the phthalate/DINCH measurements from Riksmaten adolescents, for which only limits of detection (LODs) were reported. Chemical measurements below the LOQ (or below the LOD for phthalate/DINCH from Riksmaten adolescents) were imputed by single random imputation from a truncated lognormal distribution within each study. Only chemicals that were detected in at least 70% of the samples of each study were considered, resulting in the inclusion of three out of 12 PFAS (perfluorooctanoic acid (PFOA), perfluorooctane sulfonic acid (PFOS), perfluorohexane sulfonic acid (PFHxS)), 8 out of 15 phthalate metabolites (mono-benzyl phthalate (MBzP), mono(2-ethylhexyl) phthalate (MEHP), mono(2-ethyl-5-hydroxyhexyl) phthalate (5OH-MEHP), mono(2-ethyl-5oxo-hexyl) phthalate (5oxo-MEHP), mono(2-ethyl-5-carboxypentyl) phthalate (5cx-MEPP), mono-ethyl phthalate (MEP), OH-(mono-isononyl) phthalate (OH-MiNP), carboxy-(mono-isononyl) phthalate (cx-MiNP]), and 2 DINCH components (hydroxy-mono-(isononyl) cyclohexane-1,2-dicarboxylate (OH-MINCH), carboxy-mono-(isononyl) cyclohexane-1,2-dicarboxylate (cx-MINCH)) (Supplemental Table S1). PFOS concentrations in FLEHS IV could be an underestimation, as only the linear form was measured, while the other studies included the branched forms that may contribute 30–42% of the total PFOS load in serum [59]. A total of 15 out of 542 participants (2.8%) were excluded because of missing exposure data (8 from PCB cohort follow-up, 7 from GerES V-sub).

### 2.3. Outcome and Covariates

Age at menarche (in complete years) was obtained from questionnaires (Supplemental Table S2). Of the 527 female teenagers having reached menarche and with complete exposure data, 6 (1.1%) were excluded because of missing info on age at menarche (1 from Riksmaten adolescents 2016–2017, 3 from GerES V-sub, and 2 from FLEHS IV). Height and weight of participants were measured by trained field staff in all four studies. A BMI z-score was calculated through standardization by age and sex according to growth charts from the World Health Organization, using the "anthroplus" package in R [60]. Participant age and highest educational level of the household were obtained from questionnaires. The classification of highest educational level of the household was based on the International Standard Classification of Education (ISCED) developed by the United Nations Educational, Scientific and Cultural Organization (UNESCO). Low education was defined as no secondary to lower secondary education (ISCED level 0–2), medium education as having attained upper secondary to post-secondary non-tertiary education (ISCED level 3–4), and high education as having attained tertiary education or higher (ISCED level ≥5). Covariate information was missing for 7 (4.5%) participants of the PCB cohort follow-up, resulting in a final sample size of 514 (129 from Riksmaten adolescents 2016–2017, 144 from the PCB cohort follow-up, 107 from GerES V-sub, and 134 from FLEHS IV).

### 2.4. Statistical Analysis

To describe the correlations between pollutant concentrations, pairwise Spearman rank correlations were calculated. The association between age at menarche and pollutant concentrations was assessed through four statistical methods: multiple linear regression (MLR), elastic net (ENET) [61], Bayesian model averaging using Bayesian adaptive sampling (BAS) [62], and Bayesian kernel machine regression (BKMR) [63]. Pollutant concentrations were ln-transformed and scaled, and all models included the following covariates: study (Riksmaten adolescents 2016–2017, PCB cohort follow-up, GerES V-sub, FLEHS IV), age (in months), BMI z-score, and highest educational level of the household (ISCED scale: low, medium, high). These covariates were selected by the work package on data management and analyses of the HBM4EU studies based on the current literature regarding risk factors for sexual maturation and determinants of phthalate and PFAS exposure. Highest educational level of the household was used as a proxy for socio-economic status (SES), which is a known determinant of puberty onset. Although the historical decrease in age at menarche has been attributed to improved social conditions, including nutritional status [64], recent studies suggest that earlier age of menarche is associated with lower SES [64,65]. For PFAS, ethnicity and breastfeeding were part of the minimal adjustment set, but were not included in the statistical analyses because this information was not available for all cohorts. Covariates were forced into the model by not penalizing them (ENET) or by including them in the minimal model (BAS/BKMR). Estimated regression coefficients were presented as the expected change in age at menarche (years) per interquartile fold change in pollutant concentrations (IQFc; the fold change of the 75th percentile over the 25th percentile in exposure), with 95% confidence intervals (CI).

We used single- as well as multi-pollutant MLR models, by entering concentrations of different pollutants in separate models and in the same model, respectively. Collinearity in the multi-pollutant MLR was assessed by estimating variance inflation factors (VIFs), with a VIF greater than five considered to indicate a problem of collinearity [66]. ENET is a hybrid penalized regression method that combines the regularization of both lasso and ridge regression [67]. Alpha was set at 0.5, and the optimal degree of penalization was determined by minimization of 10-fold cross-validation error, followed by stability selection to allow finite sample control of error rates. R packages glmnet [68] and stabsel [69] were used for ENET analysis and stability selection, respectively. For Bayesian model averaging, we used the BAS algorithm as described in Clyde et al. [62]. Unlike Markov chain Monte Carlo, BAS is guaranteed to enumerate the space of models if the number of iterations is equal to the dimension of the model space, while it transitions to a stochastic sampling algorithm when enumeration is not feasible [62]. In this analysis, we used enumeration to explore all possible different regression models (2^p^ with p the number of exposure biomarkers = 8192 models). We calculated the marginal variable inclusion probabilities (PIPs) for each exposure biomarker using the posterior sampling probabilities for each model in which they were included. The median probability model, defined as the model consisting of those variables whose PIP is at least 0.5, is often the optimal predictive model, so a PIP threshold of 0.5 is typically used as a cut-off for variable selection [70]. The R package BAS was used to implement the analysis, using the Jeffreys-Zellner-Siow prior for the regression coefficients and a uniform (flat) prior on the model space [71]. Estimates and 95% Bayesian credible intervals were obtained using the full posterior distribution of all regression coefficients. Finally, BKMR is a non-parametric method that models the exposure response using a kernel function that considers potential interactions between exposures and a possible nonlinear association between exposure and outcome [63]. We used component-wise variable selection and a Gaussian kernel function. The model was fit by running the Markov chain Monte Carlo (MCMC) sampler for 50,000 iterations, using the bkmr package in R [72]. Like for BAS, the PIPs provide a measure of variable importance, with a threshold of 0.5 typically used to identify important exposures. The main advantage of ENET and BAS models is that they run relatively quickly, even with large datasets and many substances. A disadvantage is that they do not allow for non-linear effects or interactions without further modifications. In addition, ENET provides no information regarding the precision of effect estimates. BKMR is the most flexible method in the sense that it allows for nonlinear associations and interactions between substances, but it is computationally intensive and requires critical evaluation of model convergence.

All analyses were performed in R version 4.3.0 (R Foundation for Statistical Computing, Vienna, Austria).

## 3. Results

The age ranges of female teenagers included in this study were 12–17 years for Riksmaten adolescents 2016–2017, 15–17 years for the PCB cohort follow-up, 12–18 years for GerES V-sub and 14–16 years for FLEHS IV. The median (P25–P75) age across studies was 15 (14–16) years (Table 1). The median (P25–P75) age at menarche in the pooled data was 13 (12–14) years and was slightly higher in the PCB cohort follow-up (14 years) and slightly lower in the Riksmaten adolescents 2016–2017 and GerES V-sub studies (12 years). BMI was comparable in the different studies, with a median (P25–P75) of 21.3 (19.4–23.8) kg/m^2^ across studies. The distribution of highest educational level of the household was similar for Riksmaten adolescents 2016–2017, GerES V-sub, and FLEHS IV, with the percentage medium educated ranging between 30.8 and 38.8%, and the percentage highly educated between 53.7 and 60.7%. In the PCB cohort follow-up, however, the percentages medium and highly educated were 77.1 and 15.3%, respectively.

In all four studies, the highest median PFAS concentrations were observed for PFOS (2.16 μg/L) and the lowest for PFHxS (0.34 μg/L) (Table 2). PFAS concentrations for the PCB cohort follow-up were lower, whereas phthalate concentrations were mostly higher compared to other studies. The highest median phthalate concentrations across studies were observed for MEP (52.14 μg/L), 5cx-MEPP (11.07 μg/L), and 5OH-MEHP (9.75 μg/L). Median OH-MINCH and cx-MINCH concentrations were 1.32 and 1.01 μg/L and were highest in the PCB cohort follow-up (2.48 μg/L) and FLEHS IV (1.23 μg/L), respectively. Spearman correlations above 0.5 were observed between PFOA, PFOS, and PFHxS, between MEHP, 5OH-MEHP, and 5oxo-MEHP, between 5oxo-MEHP and 5cx-MEPP, between MEHP and OH-MiNP, between 5OH-MEHP and OH-MiNP, between OH-MiNP and cxMiNP, and between OH-MINCH and cx-MINCH (Supplemental Figure S1).

**Figure 1:**
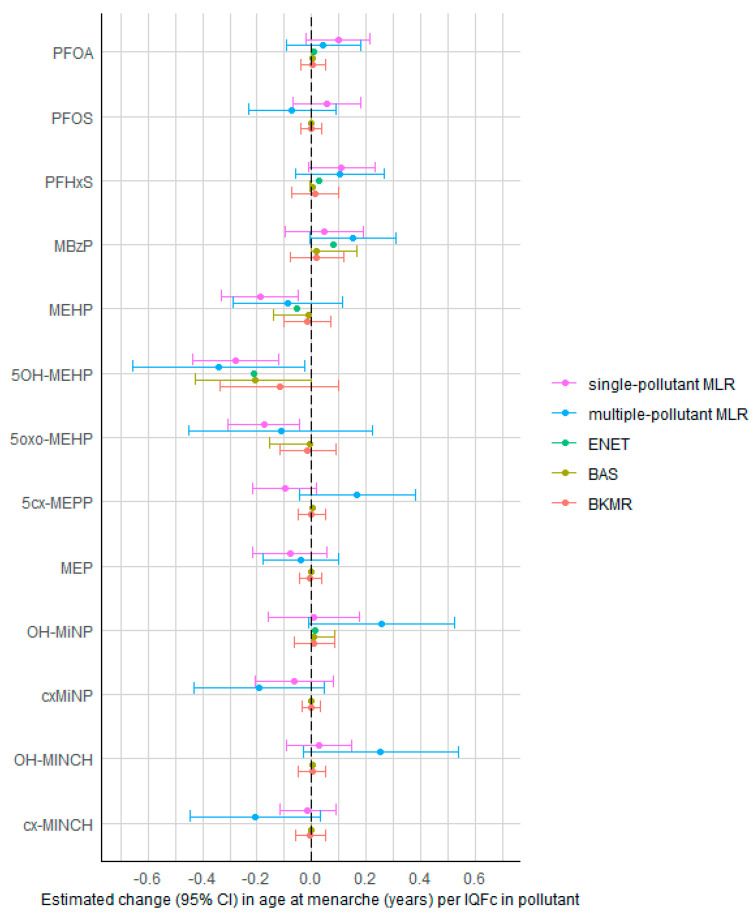
Associations between PFAS concentrations (in serum/plasma) and phthalate/DINCH concentrations (in urine) and age at menarche in female teenagers (12–18 years) of the HBM4EU aligned studies, estimated by the different statistical methods. Estimates (with 95% CI) represent the change in age at menarche (years) per interquartile fold change (IQFc) in chemical concentrations, adjusted for other chemicals (except in the single-pollutant MLR), study, age, BMI z-score, and highest educational level of the household. BKMR results are the estimates obtained when fixing other chemicals at their median value. Abbreviations: MLR = multiple linear regression; ENET = elastic net; BAS = Bayesian model averaging using Bayesian adaptive sampling; BKMR = Bayesian kernel machine regression.

In single-pollutant MLR models, increased concentrations of MEHP, 5OH-MEHP, and 5oxo-MEHP were significantly associated with lower age at menarche (Figure 1, Supplemental Table S3). The estimated age at menarche per IQFc in MEHP, 5OH-MEHP, and 5oxo-MEHP was lower by 0.19 (95% CI: 0.05, 0.33), 0.28 (95% CI: 0.12, 0.43) and 0.18 (95% CI: 0.04, 0.31) years, respectively. Other exposure biomarkers were not significant, although there was a trend of higher concentrations of PFOA and PFHxS being associated with higher age at menarche (*p*-value *<* 0.1). In the multi-pollutant MLR model, only 5OH-MEHP was significantly associated with age at menarche, with 0.34 (95% CI: 0.03, 0.66) years lower age at menarche per IQFc. A suggestive negative association was also observed for cx-MINCH, whereas suggestive positive associations were observed for MBzP, OH-MiNP, and OH-MINCH (*p*-value < 0.1). Due to the high correlation between some of the pollutants, the multi-pollutant MLR suffered from collinearity, as indicated by the wide CIs and large VIFs. VIFs were >5 for 5OH-MEHP (7.7), 5oxo-MEHP (8.0), OH-MINCH (6.2), and cx-MINCH (5.7).

In the ENET-based stability selection, no exposure met the threshold of stability selection testing with a per-family error rate (PFER) value of 0.50 and a selection probability of 0.80 (Supplemental Figure S2). For the BAS analysis, the model with only 5OH-MEHP was selected as the model with highest posterior probability, with an estimated 0.21 (95% CI: 0.00, 0.43) years lower age at menarche per IQFc in 5OH-MEHP. 5OH-MEHP was the only biomarker meeting the PIP threshold of 0.5 in the BAS model (PIP = 0.71) as well as in the BKMR model (PIP = 0.63). The BKMR model showed no evidence for interaction effects between pollutants (Supplemental Figure S3). Setting other pollutants at their median value, the estimated lower age at menarche per IQFc in 5OH-MEHP was −0.12 (95% CI: −0.33; 0.10) years.

## 4. Discussion

In this cross-sectional study, we used different statistical methods to investigate the association between PFAS and phthalate/DINCH concentrations and age at menarche in 514 teenagers from four HBM4EU aligned studies. 5OH-MEHP was most consistently associated with age at menarche according to the different modeling approaches, with significant negative effect estimates and/or a PIP value above 0.5 observed in single- and multiple-pollutant MLR, BAS, and BKMR models. In the ENET-based stability, however, 5OH-MEHP did not meet the threshold of stability selection (using a PFER value of 0.50 and a selection probability of 0.80). The estimated lower age at menarche per IQFc in 5OH-MEHP ranged from 0.12 years (BKMR) to 0.34 years (multiple-pollutant MLR). MEHP and 5oxo-MEHP concentrations were also associated with lower age at menarche in single-pollutant MLR models, likely due to their high correlation 5OH-MEHP, as these did not appear to be important predictors in the multi-pollutant models. We did not observe associations for other phthalate/DINCH biomarkers, nor for PFAS biomarkers.

### 4.1. Epidemiological Evidence for Phthalates

Modeling menarche [45,73,74] or early onset of menarche [75] as a binary outcome, four previous studies reported increased odds of (early) menarche in relation to phthalate exposure, which is consistent with our findings. In an 18-month follow-up study of 208 girls (6 to 13 years) from Shanghai, 5OH-MEHP and 5oxo-MEHP were associated with a 70% increase in the odds of having reached menarche [45]. A birth cohort study in Mexico City (8–13 years) reported suggestive associations between in utero as well as peripubertal MEP concentrations and increased odds of menarche [73,74]. In 236 girls from the Korean National Environmental Health Survey (12–17 years), the risk of early menarche was significantly higher with higher concentrations of mono-n-butyl phthalate (MnBP) and total phthalates, but no significant associations were found for age at menarche in linear regression models [75]. Other studies, however, observed later menarche in association with phthalate exposure [76,77,78], or did not find a significant association [79,80,81]. Prospective studies in Germany [78] and New York [77] reported negative associations between menarche onset and pre-pubertal phthalate exposure, more specifically for MEP and mono-hydroxy-n-butyl phthalate (OH-MnBP) and for mono-3-carboxypropyl phthalate (MCPP), respectively. In a longitudinal cohort study in California, the sum of urinary metabolites of DEHP in pregnant women was associated with later menarche in their children [76]. One study reported positive associations for DEHP metabolites measured in childhood (MEHP, 5OH-MEHP and 5oxo-MEHP) and a negative association for monomethyl phthalate (MMP) measured in adolescence [82], suggesting that discordance between studies may be due to differences in the timing of biomarker measurements. A meta-analysis including three of the above studies with risk measures for age at menarche yielded no significant association for any of the investigated phthalate metabolites (MEP, MMP, MnBP, MEHP, 5OH-MEHP, and 5oxo-MEHP) [43]. Inconsistencies between study results may be due to differences in study designs (cross-sectional or longitudinal), differences in age ranges (corresponding to different stages of development and potential differences in susceptibility) and studied phthalate metabolites (as different metabolites can exert opposite health effects [17].

### 4.2. Epidemiological Evidence for PFAS

While only a limited number of studies have investigated the association between PFAS concentrations and age at menarche, existing evidence suggests that increased PFAS exposure is associated with a delay in onset of menarche [33]. Lopez-Espinosa et al. (2011) investigated the association between PFOS and PFOA exposure and markers of puberty in 2931 girls aged 8–18 years exposed to PFOA water contamination from the Mid-Ohio Valley. Both PFOS and PFOA were significantly associated with a reduced odds of having reached puberty (based on either estradiol > 20 pg/mL or onset of menarche), but significance for PFOS disappeared after adjustment for BMI or height [51]. Similarly, a case–control study examining the influence of PFOA on hormonal endometrial regulation in 18- to 21-year-old girls found that age at menarche was significantly higher in girls from the PFAS hotspot in Veneto (*n* = 146) than in the non-exposed control group (*n* = 1080) [83]. A delayed age at menarche has also been found in association with prenatal PFOA exposure [47]. Data from the Avon Longitudinal Study of Parents and Children (ALSPAC) cohort, however, showed no associations between prenatal PFAS exposures and age at menarche [84,85]. Similarly, PFAS concentrations measured during mid-childhood were not associated with age at menarche in adolescent girls of the Project Viva cohort, although this study did find a lower pubertal development score and older age at peak height velocity in association with PFAS exposure [50].

### 4.3. Experimental Evidence and Plausible Mechanisms of Action

Severe adverse effects of phthalates on reproductive health and development have been observed in experimental studies, most consistently for DEHP, dibutyl phthalate (DBP) and benzyl butyl phthalate (BBP) [86]. In line with our results, female Wistar rats exposed to DEHP (4 weeks, 1000 mg/kg/day) had an accelerated vaginal opening indicating earlier puberty [87]. DEHP exposure (20 µg/kg/day, 200 µg/kg/day, 500 mg/kg/day) also caused earlier onset of puberty in three generations of female CD-1 mice [88]. Female marmosets orally exposed to DEHP (65 weeks, ≥500 mg/kg bw/day) showed higher estradiol concentrations and earlier puberty onset [89]. The age at vaginal opening and first estrus decreased in prepubertal female Wistar-Imamichi rats exposed to DEHP via inhalation (three treatment groups: not exposed, 5 mg/m^3^ and 25 mg/m^3^) [90]. Moreover, estrus cycles were much more irregular in the highest exposed group. Conversely, a delay in vaginal opening was observed in female Wistar rats exposed to DEHP from gestation day 6 to lactation day 22 with doses ≥ 15 mg/lg bw/day [91]. Additionally, at 135 and 405 mg/kg bw/day, a trend for a delay in age at first estrus was also observed. Female Wistar rats exposed to 70 and 700 mg/kg/day DEHP and 500 mg/kg/day DBP in utero and during lactation had delayed vaginal opening [92]. Similarly, prenatal exposure of female Sprague Dawley CD rats to 500 mg/bw BBP (from day 10 post-conception to delivery) delayed vaginal opening [93]. Regarding PFAS, there is some evidence for a delay in age at vaginal opening associated with exposure to PFOA, PFBS and PFNA in mice [94,95,96,97], while other studies found no association between PFAS exposure (PFHxA, PFHxS and PFDOA) and timing of vaginal opening [98,99]. Similarly, PFAS (PFBS, PFOA) was associated with a delay of the first estrus in some studies [94,97], but not in others [98,100].

Epidemiological and experimental evidence is supported by an internal effect of PFAS and phthalates on the central nervous system [17,101], where they can interact with the hypothalamic estrogen receptor (ER), altering the estrogenic positive and negative feedbacks on the gonadotrophin-releasing hormone (GnRH), mediated by the kisspeptinergic neurons located at the anteroventral periventricular (AVPV) and arcuate (ARC) nucleus [102]. Nevertheless, these compounds could also alter the HPG axis externally at the ovary by impairing estradiol secretion, triggering the positive and negative feedback, which is also a plausible mechanism [103,104]. The interaction with ER-mediated positive and negative feedback could alter age at menarche in both directions. If kisspeptinergic neurons from AVPV are stimulated, the positive feedback is triggered, and GnRH is secreted, upregulating pituitary and steroid hormones and triggering the sexual maturation process. If kisspeptinergic neurons from ARC are stimulated, the negative feedback is triggered, with the subsequent down-regulation of GnRH secretion, delaying puberty [102], as previously found in several experimental studies [105,106,107,108]. Longitudinal epidemiological studies including data on kisspeptin and pituitary and steroid hormones as well as information on puberty onset are needed to confirm these hypotheses. 

## 5. Strengths and Limitations

A strength of our study is the use of harmonized data collected within the HBM4EU project, enabling a pooled analysis of multiple European studies covering Northern, Eastern and Western Europe. This increases the generalizability of our study within the European context. The common set of mostly quality-assured chemical measurements included different PFAS and phthalate metabolites, including two alternative phthalate metabolites (DINCH). In contrast to most of the previous studies, we applied different statistical multi-pollutant methods, thereby accounting for the presence of correlated co-exposures. A limitation of this study is that biomarker concentrations were measured after the event (outcome), which was ascertained retrospectively. Moreover, age at menarche in complete years is only a rough indicator but gathering a more precise assessment via questionnaires might be more subject to recall bias. The observed associations may suffer from reverse causality because of physiologic or behavioral changes associated with puberty that may lead to changes in EDC concentrations in serum or urine. Menstrual blood loss is a potential route of PFAS excretion, so differences in PFAS concentrations between participants may be the consequence rather than the cause of differences in age at menarche [33,109,110]. Another limitation is the potential for exposure misclassification, especially for phthalates, which are metabolized and excreted quickly. Although single-spot urine measurements reflect recent rather than long-term exposures, longitudinal studies of EDC concentrations suggest that spot measurements are quite representative for average exposure due to recurrent exposures from common, quotidian sources [111]. Furthermore, our study was limited to age at menarche as a single marker for pubertal timing in girls. Given the only moderate correlation between menarche and onset of puberty [112,113], effects of EDCs on other sexual maturation markers may be different, which may explain inconsistencies in results observed for different pubertal timing outcomes in the literature. Finally, given the concurrence of EDCs in consumer products and the home environment, we cannot exclude the possibility of unmeasured or residual confounding by other exposures such as persistent organic pollutants, or by other covariates such as ethnicity, breastfeeding, and SES. SES of teenagers may depend on a combination of educational, financial, social, and cultural resources, so the inclusion of variables such as income of the household and parental occupation or a composite family affluence scale would have been preferred. Unfortunately, the only common variable available in the four studies was educational level (ISCED).

## 6. Conclusions

We found urinary DEHP concentrations, particularly 5OH-MEHP, to be associated with an earlier age at menarche in 12- to 18-year-old teenagers participating in the HBM4EU project, suggesting that EDCs may affect sexual maturation at concentrations detected in the general European population.

## Figures and Tables

**Table 1:** Population characteristics of female teenagers (12–18 years) for each of the four included HBM4EU aligned studies.

Characteristic	Riksmaten Adolescents 2016–2017	PCB Cohort Follow-Up	GerES V-Sub	FLEHS IV	Pooled Cohorts
Country (Region)	Sweden (North)	Slovakia (East)	Germany (West)	Belgium (West)	NA
Sampling years	2016–2017	2019–2020	2015–2017	2017–2018	2015–2020
N included	129	144	107	134	514
Age (years)					
median	14	16	15	14	15
P25–P75	14–17	15–16	14–16	14–15	14–16
BMI (kg/m^2^)					
median	21.7	21.1	21.0	21.4	21.3
P25–P75	19.7–23.9	18.9–24.1	19.3–22.7	19.5–23.6	19.4–23.8
Age at menarche (years)					
median	12	14	12	13	13
P25–P75	12–13	13–15	12–13	12–13.8	12–14
Household education (%)					
low (ISCED 0–2)	9.3	7.6	8.4	7.5	8.2
medium (ISCED 3–4)	31.0	77.1	30.8	38.8	45.9
high (ISCED 5–8)	59.7	15.3	60.7	53.7	45.9

Abbreviations: P25 = 25th percentile; P75 = 75th percentile; ISCED = International Standard Classification of Education.

**Table 2:** PFAS and phthalate/DINCH concentrations measured in female teenagers (12–18 years) for each of the four included HBM4EU aligned studies.

Biomarker	Riksmaten Adolescents 2016–2017	PCB Cohort Follow-Up	GerES V-Sub	FLEHS IV	Pooled Cohorts
PFAS in serum/plasma (μg/L), median (P25–P75)					
PFOA	1.14 (0.90–1.56) *	0.66 (0.45–0.88)	1.17 (0.70–1.75)	1.00 (0.81–1.30)	0.96 (0.68–1.30)
PFOS	2.55 (1.78–3.71) *	1.22 (0.76–2.29)	2.41 (1.73–3.39)	2.10 (1.40–3.35)	2.16 (1.30–3.05)
PFHxS	0.36 (0.26–0.51) *	0.25 (0.18–0.33)	0.35 (0.22–0.47)	0.45 (0.32–0.61)	0.34 (0.22–0.52)
Phthalates in urine (μg/L), median (P25–P75)					
MBzP	8.63 (3.38–16.27)	1.89 (1.01–4.09)	2.40 (1.59–4.71)	2.28 (1.23–5.56)	2.80 (1.49–7.10)
MEHP	1.81 (1.33–2.96)	5.25 (2.77–9.00)	1.54 (0.91–2.69)	1.39 (0.84–2.33)	2.01 (1.17–4.17)
5OH-MEHP	7.47 (5.39–12.41)	46.66 (23.73–85.97)	8.32 (5.86–11.51)	6.28 (3.61–9.67)	9.75 (5.89–23.70)
5oxo-MEHP	5.84 (4.21–9.95)	11.09 (5.84–15.92)	5.87 (3.78–8.22)	4.13 (2.40–6.39)	6.04 (3.77–11.16)
5cx-MEPP	6.84 (4.62–12.02)	12.60 (7.51–21.77)	8.19 (5.78–13.21)	16.15 (11.27–22.47)	11.07 (6.78–17.85)
MEP	52.14 (27.88–104.18)	89.79 (45.85–192.35)	30.63 (15.80–122.02)	32.13 (17.02–77.69)	52.14 (21.62–122.36)
OH-MiNP	3.77 (2.38–7.32)	25.66 (15.28–44.13)	5.85 (4.11–9.07)	4.03 (2.69–5.62)	6.31 (3.43–16.66)
cxMiNP	6.78 (3.81–12.52)	9.97 (6.03–18.14)	4.80 (3.17–7.61)	1.88 (1.27–2.75)	5.07 (2.67–10.62)
DINCH in urine (μg/L), median (P25–P75)					
OH-MINCH	0.85 (0.44–1.53)	2.48 (1.18–4.03)	1.30 (0.81–2.45)	1.22 (0.72–2.38)	1.32 (0.72–3.01)
cx-MINCH	0.89 (0.52–1.82)	1.11 (0.60–2.11)	0.76 (0.45–1.31)	1.23 (0.79–1.81)	1.01 (0.58–1.74)

Abbreviations: PFOA = perfluorooctanoic acid; PFOS = perfluorooctane sulfonic acid (only the linear form in FLEHS IV, sum of all isomers in the other studies), PFHxS = perfluorohexane sulfonic acid; MBzP = mono-benzyl phthalate; MEHP = mono(2-ethylhexyl) phthalate; 5OH-MEHP = mono(2-ethyl-5-hydroxyhexyl) phthalate; 5oxo-MEHP = mono(2-ethyl-5oxo-hexyl) phthalate; 5cx-MEPP = mono(2-ethyl-5-carboxypentyl) phthalate; MEP = mono-ethyl phthalate; OH-MiNP = OH-(mono-isononyl) phthalate; cxMiNP = carboxy-(mono-isononyl) phthalate; OHMINCH = hydroxy-mono-(isononyl) cyclohexane-1,2-dicarboxylate; cx-MINCH = carboxy-mono-(isononyl) cyclohexane-1,2-dicarboxylate. All studies measured PFAS concentrations in serum and phthalates and DINCH in spot urine, except for GerES V-sub, which provided plasma and morning urine measurements, respectively. * PFAS concentrations in Riksmaten adolescents were reported in µg/kg. To convert them to µg/L, the assumption was made that 1 mL blood serum = 1 g blood serum.

## Data Availability

The data that have been used are subject to the GDPR (EU 2016/679, “General Data Protection Regulation”). Hence, the data are not openly accessible but can be requested via https://hbm.vito.be (accessed on 13 June 2023).

## Supplementary Materials

The following supporting information can be downloaded at: https://www.mdpi.com/article/10.3390/toxics11080711/s1, Table S1: Limits of quantification or detection and percentage of the samples below this limit for PFAS and phthalate/DINCH measurements in teenagers (12–18 years) for each of the four included HBM4EU aligned studies; Table S2: Exact phrasing and answer options for the questions on menarche and age at menarche in the questionnaires from the different studies; Table S3: Associations between PFAS concentrations (in serum/plasma) and phthalate/DINCH concentrations (in urine) and age at menarche in female teenagers (12–18 years) of the HBM4EU aligned studies, estimated by the different statistical methods; Figure S1: Spearman correlations between the different pollutant biomarkers measured in female teenagers (12–18 years) of the four included HBM4EU aligned studies (*n* = 514); Figure S2: Stability selection for elastic net; Figure S3: Associations between age at menarche and PFAS and phthalate/DINCH concentrations in female teenagers (12–18 years) of the HBM4EU aligned studies, estimated by the BKMR model.
