# Supplementary material for: PFAS and Phthalate/DINCH Exposure in Association with Age at Menarche in Teenagers of the HBM4EU Aligned Studies
Source: Toxics. 2023 Aug 18;11(8):711. doi: 10.3390/toxics11080711 (PMC10459167; doi:10.3390/toxics11080711)
Supplement: Supplementary file 1 [file toxics-11-00711-s001.zip › toxics-2493844-supplementary.pdf]

Supplementary materials

PFAS and Phthalate/DINCH Exposure in Association with Age at Menarche in Teenagers of the HBM4EU Aligned Studies

Contents

Table S1. Limits of quantification or detection and percentage of the samples below this limit for PFAS and phthalate/DINCH measurements in teenagers (12–18 years) for each of the four included HBM4EU aligned studies. 2

Table S2. Exact phrasing and answer options for the questions on menarche and age at menarche in the questionnaires from the different studies. 3

Figure S1. Spearman correlations between the different pollutant biomarkers measured in female teenagers (12–18years) of the four included HBM4EU aligned studies (n=514). 4

Table S3. Associations between PFAS concentrations (in serum/plasma) and phthalate/DINCH concentrations (in urine) and age at menarche in female teenagers (12–18 years) of the HBM4EU aligned studies, estimated by the different statistical methods........ 5

Figure S2. Stability selection for elastic net. Selection frequencies for the different pollutant biomarkers, using a threshold of 0.8 for the selection frequency and targeting a per-family error rate (PFER) of 0.5. 6

Figure S3. Associations between age at menarche and PFAS and phthalate/DINCH concentrations in female teenagers (12–18 years) of the HBM4EU aligned studies, estimated by the BKMR model. 7

Table S1. Limits of quantification or detection and percentage of the samples below this limit for PFAS and phthalate/DINCH measurements in teenagers (12–18 years) for each of the four included HBM4EU aligned studies.

Biomarker	Riksmaten adolescents 2016-17			PCB cohort follow-up			GerES V-sub			FLEHS IV		
	LOQ/LOD[a]	%<LOQ/LOD		LOQ	%<LOQ		LOQ	%<LOQ		LOQ	%<LOQ	
		Original[b]	Final[c]		Original[b]	Final[c]		Original[b]	Final[c]		Original[b]	Final[c]
PFAS in serum/plasma (µg/L)		n=300	n=129		n=292	n=144		n=300	n=107		n=300	n=134
PFOA	0.020-0.288	0	0	0.014	0.34	0.69	0.5	13.67	19.27	0.2	0	0
PFOS	0.056-0.562	0	0	0.04	0	0	0.25	0	0	0.2	0	0
PFHxS	0.022-0.464	11	15.5	0.016	0.34	0.69	0.25	27.67	33.94	0.2	2.33	5.22
Phthalates in urine (µg/L)		n=300	n=129		n=287	n=144		n=291	n=107		n=300	n=134
MBzP	0.200	0.33	0.78	0.3	8.36	9.03	0.2	0.34	0	0.2	1.67	2.99
MEHP	0.300	2	2.33	0.5	2.09	2.78	0.5	13.06	15.6	0.5	18.67	24.63
5OH-MEHP	0.100	0	0	0.2	0.35	0.69	0.2	0	0	0.2	0.33	0.75
5oxo-MEHP	0.200	0	0	0.2	0	0	0.2	0	0	0.2	0.67	1.49
5cx-MEPP	0.070	0	0	0.2	0.7	1.39	0.2	0	0	0.5	0	0
MEP	0.200	0	0	0.6	0	0	0.5	0	0	0.5	0	0
OH-MiNP	0.050	0	0	0.7	0	0	0.2	0	0	0.2	0	0
cxMiNP	0.050	0	0	0.4	1.05	2.08	0.2	0	0	0.2	0.33	0.75
DINCH in urine (µg/L)		n=300	n=129		n=287	n=144		n=291	n=107		n=300	n=134
OHMINCH	0.100	2.33	4.65	0.14	0	0	0.05	0	0	0.2	4.33	5.22
cx-MINCH	0.100	2	3.1	0.1	0.7	1.39	0.05	1.37	0.92	0.2	1.67	2.24

Abbreviations: LOQ=limit of quantification; LOD=limit of detection; PFOA=perfluorooctanoic acid; PFOS=perfluorooctane sulfonic acid (only the linear form in FLEHS IV, sum of all isomers in the other studies), PFHxS=perfluorohexane sulfonic acid; MBzP=mono-benzyl phthalate; MEHP=mono(2-ethylhexyl) phthalate; 5OH-MEHP=mono(2-ethyl-5-hydroxyhexyl) phthalate; 5oxo-MEHP=mono(2-ethyl-5oxo-hexyl) phthalate; 5cx-MEPP=mono(2-ethyl-5-carboxypentyl) phthalate; MEP=mono-ethyl phthalate; OH-MiNP=OH-(mono-isononyl) phthalate; cxMiNP=carboxy-(mono-isononyl) phthalate; OHMINCH=hydroxy-mono-(isononyl) cyclohexane-1,2-dicarboxylate; cx-MINCH=carboxy-mono-(isononyl) cyclohexane-1,2-dicarboxylate. Chemicals were measured by liquid chromatography-tandem mass spectrometry (LC-MS/MS), except for PFAS data from Riksmaten adolescents 2016-17, which were measured by ultraperformance liquid chromatography-tandem mass spectrometry (UPLC-MS/MS). Chemical measurements were rated quality assured by the HBM4EU quality assurance quality control (QA/QC) program [55,56], except for OH-MiNP measurements from the PCB cohort follow-up and for PFAS and MEP measurements from Riksmaten adolescents 2016-17. All studies measured PFAS concentrations in serum and phthalates and DINCH in spot urine, except for GerES V-sub, which provided plasma and morning urine measurements, respectively. PFAS-concentrations in Riksmaten adolescents were reported in µg/kg. To convert them to µg/L, the assumption was made that 1 mL blood serum = 1 g blood serum

[a] All laboratories reported LOQs, except for the phthalate/DINCH measurements from Riksmaten adolescents, for which LODs were reported. LOQs for PFAS measurements from Riksmaten adolescents are represented as minimum and maximum values.

[b] Original sample that was used for imputation of values <LOQ/LOD

[c] Final sample used in the analysis

Table S2. Exact phrasing and answer options for the questions on menarche and age at menarche in the questionnaires from the different studies.

Study	Question asking whether participants have had their first menstruation	Question asking the age at menarche
Riksmaten adolescents 2016-17	Have you had your first menstrual period? → Yes/No	How old were you when you got your first menstrual period? → Multiple choice:<br>10 years or younger<br>11 years<br>12 years<br>13 years<br>14 years<br>15 years<br>16 years<br>17 years<br>18 years
PCB cohort follow-up	/	At what age did you have your first period? → Years
GerES V-sub	Have you already had your first menstrual bleeding (period)? → Yes/No	At what age have you had your first menstrual bleeding? → Years
FLEHS IV	Have you already had your period (menstrual bleed)? → Yes/No	In case the previous question was yes: When did you first get your menstruation? → Month and Year

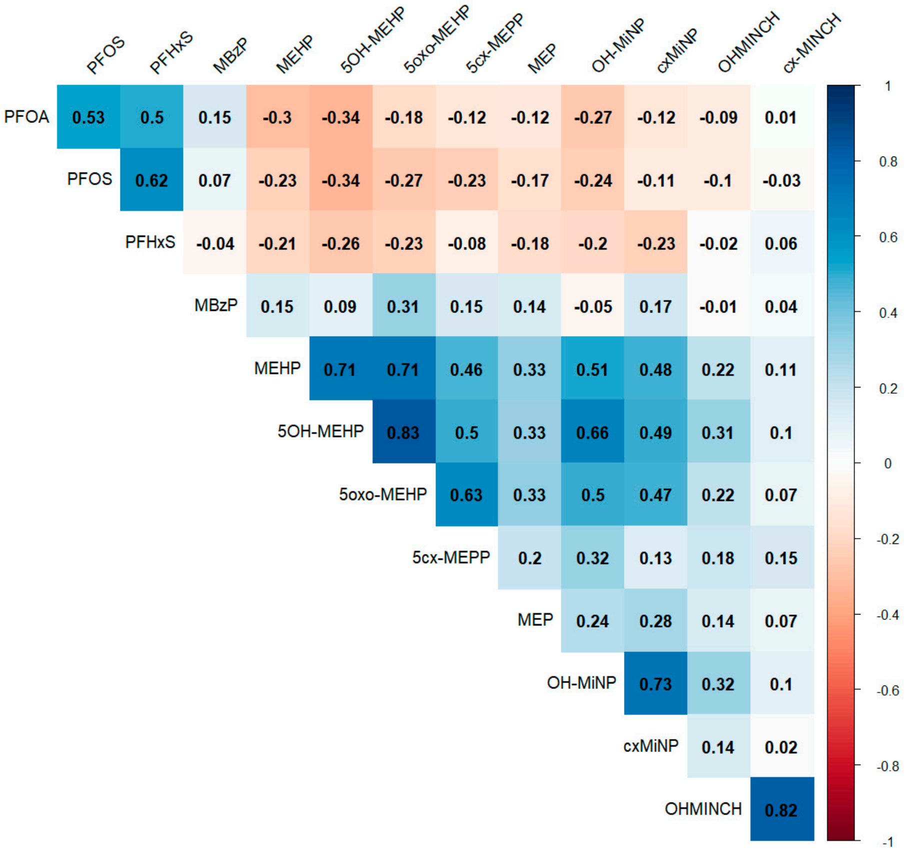

Figure S1. Spearman correlations between the different pollutant biomarkers measured in female teenagers (12–18 years) of the four included HBM4EU aligned studies (n=514).

The color intensity of the cells indicates the strength of the correlation (darker color = higher correlation), with positive correlations presented in blue and negative correlations in red.

Table S3. Associations between PFAS concentrations (in serum/plasma) and phthalate/DINCH concentrations (in urine) and age at menarche in female teenagers (12–18 years) of the HBM4EU aligned studies, estimated by the different statistical methods.

	Single-pollutant MLR		Multiple-pollutant MLR		ENET	BAS		BKMR	
	β (95% CI)	*p*-value	β (95% CI)	*p*-value	β	PIP	β (95% CI)	PIP	β (95% CI)
PFOA	0.10 (-0.02; 0.22)	0.099	0.04 (-0.09; 0.18)	0.532	0.01	0.04	0.00 (0.00; 0.00)	0.07	0.00 (-0.04; 0.05)
PFOS	0.06 (-0.07; 0.18)	0.372	-0.07 (-0.23; 0.09)	0.383	-	0.02	0.00 (0.00; 0.00)	0.09	0.00 (-0.04; 0.04)
PFHxS	0.11 (-0.01; 0.23)	0.070	0.10 (-0.06; 0.27)	0.205	0.04	0.05	0.00 (-0.01; 0.00)	0.13	0.01 (-0.07; 0.10)
MBzP	0.05 (-0.09; 0.19)	0.514	0.15 (-0.01; 0.31)	0.062	0.07	0.12	0.02 (0.00; 0.17)	0.19	0.02 (-0.08; 0.12)
MEHP	-0.19 (-0.33; -0.05)	**0.009**	-0.09 (-0.29; 0.11)	0.393	-0.05	0.08	-0.01 (-0.14; 0.00)	0.14	-0.01 (-0.10; 0.07)
5OH-MEHP	-0.28 (-0.43; -0.12)	**0.001**	-0.34 (-0.66; -0.03)	**0.034**	-0.18	**0.71**	-0.21 (-0.43; 0.00)	**0.63**	-0.12 (-0.33; 0.10)
5oxo-MEHP	-0.18 (-0.31; -0.04)	**0.010**	-0.11 (-0.45; 0.22)	0.507	-	0.07	-0.01 (-0.15; 0.00)	0.19	-0.01 (-0.12; 0.09)
5cx-MEPP	-0.10 (-0.22; 0.02)	0.105	0.17 (-0.04; 0.38)	0.121	-	0.03	0.00 (0.00; 0.00)	0.08	0.00 (-0.05; 0.05)
MEP	-0.08 (-0.21; 0.06)	0.248	-0.04 (-0.18; 0.10)	0.570	-	0.03	0.00 (0.00; 0.00)	0.07	0.00 (-0.05; 0.04)
OH-MiNP	0.01 (-0.16; 0.18)	0.918	0.26 (-0.01; 0.53)	0.061	0.01	0.06	0.01 (0.00; 0.08)	0.10	0.01 (-0.07; 0.08)
cxMiNP	-0.06 (-0.21; 0.08)	0.378	-0.19 (-0.43; 0.05)	0.118	-	0.03	0.00 (0.00; 0.00)	0.04	0.00 (-0.03; 0.03)
OHMINCH	0.03 (-0.09; 0.15)	0.666	0.25 (-0.03; 0.54)	0.081	-	0.04	0.00 (0.00; 0.00)	0.07	0.00 (-0.05; 0.05)
cx-MINCH	-0.01 (-0.12; 0.09)	0.777	-0.21 (-0.45; 0.03)	0.090	-	0.03	0.00 (0.00; 0.00)	0.09	-0.01 (-0.06; 0.05)

Estimates (with 95% CI) represent the change in age at menarche (years) per interquartile fold change (IQFc) in chemical concentrations, adjusted for other chemicals (except in the single-pollutant MLR), study, age, BMI z-score, and highest educational level of the household. BKMR results are the estimates obtained when fixing other chemicals at their median value. *P*-values < 0.05 and PIP values > 0.5 are marked in bold.
Abbreviations: MLR=multiple linear regression; ENET=elastic net; BAS=Bayesian model averaging using Bayesian adaptive sampling; BKMR=Bayesian kernel machine regression; PIP= posterior inclusion probabilities.

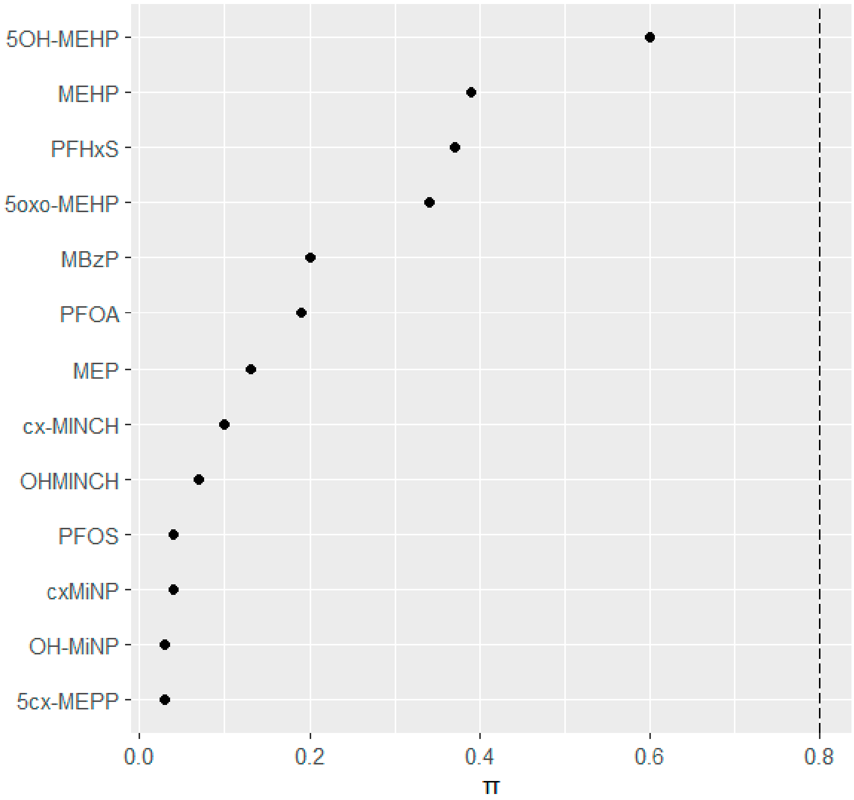

Figure S2. Stability selection for elastic net. Selection frequencies for the different pollutant biomarkers, using a threshold of 0.8 for the selection frequency and targeting a per-family error rate (PFER) of 0.5.

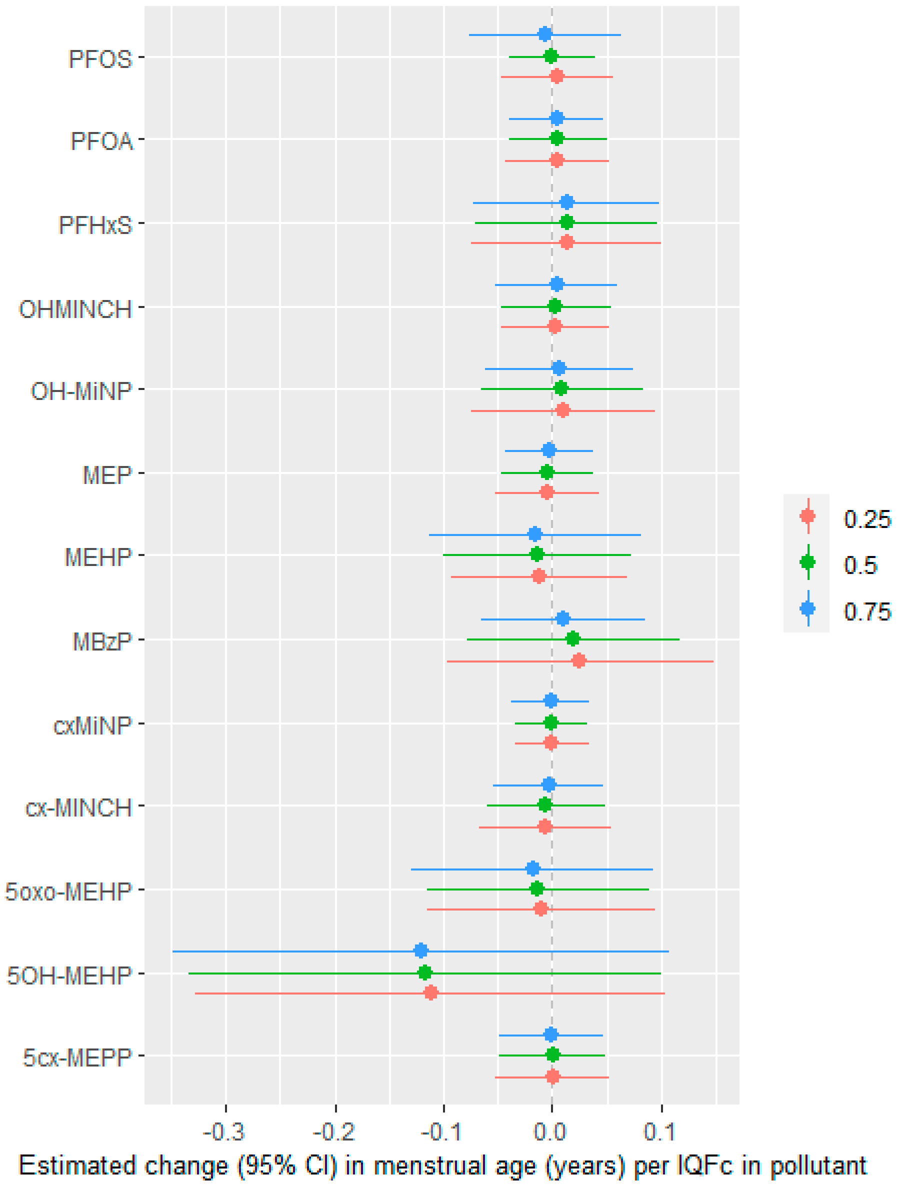

Figure S3. Associations between age at menarche and PFAS and phthalate/DINCH concentrations in female teenagers (12–18 years) of the HBM4EU aligned studies, estimated by the BKMR model.

Estimates (with 95% CI) represent the change in age at menarche (years) per interquartile fold change (IQFc) in chemical concentration, fixing other chemicals at their 25th (red), 50th (green), and 75th (blue) percentiles. Estimates are adjusted for other chemicals, study, age, BMI z-score, and highest educational level of the household.
